# Immunomodulatory Effect of Vitamin D and Its Potential Role in the Prevention and Treatment of Type 1 Diabetes Mellitus—A Narrative Review

**DOI:** 10.3390/molecules24010053

**Published:** 2018-12-24

**Authors:** Karolina Rak, Monika Bronkowska

**Affiliations:** Department of Human Nutrition, Faculty of Biotechnology and Food Science, Wrocław University of Environmental and Life Sciences, ul. Chełmońskiego 37, 51-630 Wrocław, Poland; monika.bronkowska@upwr.edu.pl

**Keywords:** vitamin D, calcitriol, immunomodulatory effect, type 1 diabetes mellitus

## Abstract

Type 1 diabetes mellitus is a chronic autoimmune disease associated with degeneration of pancreatic β-cells that results in an inability to produce insulin and the need for exogenous insulin administration. It is a significant global health problem as the incidence of this disorder is increasing worldwide. The causes are still poorly understood, although it certainly has genetic and environmental origins. Vitamin D formed profusely in the skin upon exposure to sunlight, as well as from dietary sources, exhibits an immunomodulatory effect based on gene transcription control. Indeed, vitamin D can downregulate mechanisms connected with adaptive immunity, induce immunological tolerance and decrease auto-aggression-related inflammation. These properties provide the basis for a preventive and therapeutic role of vitamin D. As many studies have demonstrated, appropriate supplementation with vitamin D reduces the risk of autoimmune diseases, including type 1 diabetes mellitus, and alleviates disease symptoms in patients. The aim of this narrative review is to present the molecular mechanisms for the vitamin D immunomodulatory effect as well as review human clinical studies on the use of vitamin D as adjuvant therapy in type 1 diabetes mellitus.

## 1. Type 1 Diabetes Mellitus—A Global Public Health Emergency

Type 1 diabetes mellitus (T1DM) is a chronic autoimmune disease characterised by degeneration of pancreatic β-cells. This action results in the inability to produce insulin and need for exogenous insulin administration. T1DM is accompanied by auto-aggression-induced inflammation. The cause of this disease remains unknown; however, there are genetic factors that increase susceptibility to the disease as well as environmental triggers [1,2]. It appears suddenly and occurs at any age, but particularly in childhood [3]. 

The prevalence of all types of diabetes is increasing worldwide and becoming a serious global public health problem. In 2014, 422 million people around the world suffered from diabetes, compared to 108 million in 1980. During this time, the incidence of diabetes increased from 4.7 to 8.5% in the adult population [4]. Between 5 to 10% of diabetes cases are T1DM. The prevalence of T1DM is also increasing by approximately 3% every year. Currently, there are 542,000 children under 14 years old with T1DM, most of whom reside in the United States, India, Brazil and China [5].

Undiagnosed or untreated diabetes of all types, including T1DM, can cause life-changing and life-threatening health complications in many organs. Chronic elevated blood glucose can lead to heart attack, stroke, nerve damage, vision loss, kidney failure or leg amputation [4]. Moreover, patients with diabetes very often have to be treated for hypertension, dyslipidemia, microalbuminuria or nephropathy [6]. Finally, diabetic complications result in premature death, estimated in 2012 at 1.5 million caused directly by diabetes and an additional 2.2 million caused indirectly by higher-than-optimal blood glucose [4]. 

Diabetes and its complications bring economic expenditures from patients and their families and the national healthcare system. These costs are connected with hospital and outpatient care, insulin and other essential medicines, loss of productivity, loss of work and wages and long-term national financial support. In most countries, 5–20% of healthcare expenditures are spent on diabetes and diabetes-related health consequences [4,5]. This fact clearly suggests that this disease is a significant challenge for healthcare systems and raises the need to search for low-cost adjuvant therapies for diabetic patients.

## 2. Genetic and Environmental Risk Factors for T1DM

T1DM is the final consequence of β-cell autoimmunity (islet autoimmunity, IA). During the first stage, autoantibodies against insulin and/or glutamic acid decarboxylase occur. Next, additional autoantibodies against islet antigen-2 or zinc transporter-8 appear [7]. The persistent presence of at least two types of β-cell autoantibodies results in irreversible IA progression to T1DM, and in 70% of IA-positive individuals, diabetes onset appears within the next 10 years [8].

The primary risk factor for IA (and subsequently T1DM) is genetic in origin as it occurs mainly in individuals with specific haplotypes of human leukocyte antigen that are involved in regulation of immune responses and recognition of self versus non-self cells: HLA-DR3-DQ2 or HLA-DR4-DQ8 [7]. Inheritance of these HLA alleles is responsible for over half of the overall T1DM genetic risk [9]. Genetic susceptibility only predisposes an individual to diabetes development, and environmental trigger(s) is/are necessary for IA and subsequent T1DM onset [10]. There is myriad evidence that supports the genetic-environmental origin of T1DM. The increasing prevalence of T1DM over the past few decades cannot result from genetic factors alone since genetic changes need much more time to appear [11]. Moreover, there is a substantial difference in T1DM incidence among countries characterised by very similar distribution and frequency of HLA genes that predispose autoimmunity [12]. T1DM incidence also varies significantly between populations with similar racial and ethnic backgrounds [13] as well as in individuals who are genetically close but separated by socioeconomic borders [14]. Further, a similar T1DM risk is commonly observed in migrants and native inhabitants despite the obvious genetic differences [15]. Finally, there is only evidence for partial T1DM genetic determination from studies that involve monozygotic twins [16].

Microbial and dietary triggers are among the environmental factors that might contribute to islet autoantibodies and consequently diabetes [10]. Both animal models and human studies suggest viral infections, especially caused by enteroviruses, are a potential trigger for T1DM [17,18,19,20,21,22]. Moreover, the intestinal commensal microbiota appear to be a potential modulator of T1DM risk since the composition of gut and fecal microorganisms is different in children with IA/T1DM compared to healthy controls [23,24]. Lower microbial diversity was suggested to increase the risk of progression to T1DM in IA-positive children [23,25]. There has been speculation about a potential association between vaccines and autoimmunity. The results of studies, however, revealed no association of vaccines with IA [26,27] or T1DM [28,29]; these findings were fully supported by a recent meta-analysis that investigated 16 vaccinations [30].

Dietary factors are an important part of the discussion about potential T1DM triggers, although results from studies seem to be contradictory. A lot of studies focused on infant feeding, especially breastfeeding, and the age of introduction of specific food groups, notably cereals and cow milk. Breastfeeding has a protective role against autoimmunisation and reduced the IA risk in 5-year-old Swedish children [31]. Moreover, children who were still breastfed during cereal introduction into the infant diet had reduced risk of IA [32] and T1DM [33]. However, prospective birth cohort studies do not support findings about the protective effect of breastmilk [34,35,36]. Introduction too early (before the fourth month of life) or too late (after the sixth month of life) of any type of cereals (gluten or gluten-free) was both associated [32] and not [37] with increased IA risk. Other studies reported a significant association only with early [35] or late [38] exposure to gluten as well as no clear association with the moment of introduction of this cereal protein [36]. Interestingly, a gluten-free diet at 6–12 month of age did not reduce the incidence of IA in infants with high genetic risk [39] and did not decrease the level of autoantibodies in IA-positive children [40].

Similarly, studies present contradictory results regarding the potential impact of cow milk intake during childhood on IA and T1DM risk. Many prospective birth cohort studies [31,32,33,34,35] and randomised clinical trials [41] found no association between early exposure to cow milk and IA or T1DM. There are, however, results that suggest cow milk introduction in the second semester of life increases the risk of developing T1DM almost 4-fold in later life [42]. Studies that investigated the role of cow milk intake in later childhood also present mixed results. Some demonstrated an association between cow milk consumption with increased risk of IA [37,38,43] and T1DM [43,44,45], while others reported a decreased risk with T1DM [46]. A recent review on this issue conducted by Chia et al. concluded that A1 β-casein from cow milk may be a significant trigger for T1DM in genetically susceptible individuals. In the authors’ opinion, many other factors modulate the risk, e.g., aberrant mucosal immunity, breastfeeding duration, exposure to other dietary triggers and vitamin D as well as timing and magnitude and/or duration of exposure to A1 β-casein [47].

Another potential dietary risk factor for IA and T1DM is high meat consumption. Ecological analysis of 37 world areas demonstrated an association between increased T1DM incidence and meat consumption, but not milk and cereal [48]. One study reported a dose-response association between meat, but no other examined food items intake in early life, and T1DM in childhood [49]. Moreover, maternal intake of red meat, especially processed meat, during lactation also apparently promotes T1DM development in the offspring [50]. *N*-nitroso compounds, preservatives commonly present in meat products, seem to be an important trigger of T1DM in children [51,52,53]. 

Vitamin D insufficiency and deficiency, as it will be detailed presented in Section 6, is a strong dietary risk factor for IA and T1DM development, especially at specific life stages. The problem of vitamin D status and T1DM risk is very complicated; it depends not only on dietary habits, supplementation intake and sunlight exposure, but also on polymorphisms in genes involved in the vitamin D metabolic pathway, including vitamin D receptor (VDR) [54,55,56,57,58,59,60,61], vitamin D binding protein (VDBP) [62,63,64,65,66,67,68], vitamin D 25-hydroxylase (CYP2R1) and 1α-hydroxylase (CYP27B1) [67,69,70,71,72].

VDR is found on the surface of almost all human cells, a distribution that enables the multidirectional impact of vitamin D. Studies on associations between VDR gene polymorphisms, namely Fok-I, Bsm-I, Apa-I and Taq-I, and T1DM susceptibility are inconsistent and inconclusive. Most of them demonstrated that Bsm-I [54,55,56,57,58] and Fok-I [54,55,56,59] increase T1DM risk. However, there is a discrepancy regarding which alleles most predispose an individual to diabetes development: B [56,57] or b [54,55] and F [55,56] or f [54]. Fok-I F and Bsm-I B less commonly occur in T1DM patients compared to healthy controls [60]. Some studies indicate that Taq-I [57,60] and Apa-I [59,60] polymorphisms increase T1DM risk, although researchers present divergent conclusions on predisposing alleles: T [60] or t [57]. The results of a comprehensive meta-analysis conducted by Tizaoui et al. showed no association between individual VDR polymorphisms and T1DM risk, but haplotypes contributed significantly to disease susceptibility. Study characteristics (e.g., publication year, age, gender, vitamin D level and latitude) moderate the association between VDR polymorphisms and T1DM. This finding suggests that interactions of VDR polymorphisms among each other and with environmental factors contribute to T1DM pathogenesis [61]. This conclusion seems to explain the inconsistent results presented by different authors. 

VDBP is the crucial systemic transporter of 1,25-dihydroxyvitamin D and is essential for its cellular endocytosis [62]. VDBP has three polymorphisms that may alter the affinity for 1,25(OH)_2_D [63] and affect the serum 1,25(OH)_2_D level [64]. Some studies report a relationship between VDBP polymorphisms and T1DM [65,66], although others did not support this finding [67,68]. T1DM patients, however, have a lower level of VDBP compared to healthy controls [68]. 

Two main enzymes are involved in the vitamin D metabolic pathway: vitamin D 25α-hydroxylase converts vitamin D to 25(OH)D, encoded by CYP2R1, and 1α-hydroxylase that converts 25(OH)D to 1,25(OH)_2_D, encoded by CYP27B1 [69]. There is an association among CYP2R1 and CYP27B1 polymorphisms and T1DM susceptibility in many [69,70,71,72], but not all studies [67]. 

Additional studies are urgently needed to clearly establish the association between polymorphisms in genes involved in the vitamin D metabolic pathway, including specific alleles, and vitamin D status. Subjects in future studies may be divided according to various alleles in the vitamin D pathway. 

## 3. Vitamin D—Sources and Recommendations

Vitamin D is a fat-soluble steroid and precursor for human steroid hormones. One molecule is composed of four rings and one side chain. There are two natural sources of this vitamin: dietary (especially D_3_ from animal food and D_2_ contained in yeast and fungi) and exposure to UVB sunlight (D_3_) [73]. Sea fish and fish oils contain high vitamin D_3_ levels (7.5–25 µg/100 g), although small amounts are also found in dairy products and eggs (0.5–2.5 µg/100 g). Dietary sources cover only approximately 20% of the recommended daily intake of this nutrient, while the vast majority of vitamin D circulating in the body comes from its biosynthesis in the skin upon exposure to UVB sunlight (75 µg/10 min exposure to direct sunlight of arms and legs) [74].

The recommended daily intake of vitamin D_3_ (AI) developed by the Endocrine Practice Guidelines Committee, due to the global vitamin D deficiency pandemic [75], is 400–1000 IU for infants, 600–1000 IU for children and adolescents aged 1–18 years and 1500–2000 IU for adults (including pregnant and lactating women) and elders [76]. Vitamin D supplementation guidelines for Central Europe suggest slightly lower doses for infants (0–6 mo—400 IU, 7–12 mo—400–600 IU) and adults (800–2000 IU) than in USA, whereas recommendations for children and adolescents aged 1–18 years remains the same [77]. Moreover, separated recommendation regarding daily intake of vitamin D for pregnant and lactating women (1500–2000 IU) has been established [77]. The latest daily vitamin D intake recommendations for the general Polish population have been however tightened: 800–2000 IU for individuals aged 11–65 years, 2000 IU for seniors (>65–75 years), 2000–4000 IU for eldest seniors (>75 years) and 2000 IU for pregnant and lactating women [78]. Additionally, children, adolescents and adults (1–65 years(s)) may supplement vitamin D by sunbathing with uncovered forearms and legs (without sunscreen) for at least 15 min between 10:00 and 15:00 from May to September. In seniors (>65 years), due to decreased efficiency of the skin synthesis, vitamin D supplementation is recommended throughout the entire year [78]. Supplementation of up to 10,000 IU vitamin D per day has been recognized as a safe and tolerable upper intake level for adults by the United States Academy of Sciences Institute of Medicine [79]. 

Interestingly, Carlberg and Hag, in their recent paper, propose the concept of the personal vitamin D response index. They suggest that the need for vitamin D supplementation depends on vitamin D status with regards to an individual’s personal vitamin D response index rather than on vitamin D status alone [80]. They claim that individuals can be distinguished into high, mid and low vitamin D responders by measuring vitamin-D-sensitive molecular parameters. 

It is recommended to evaluate vitamin D status using the serum 25(OH)D concentration and define vitamin D deficiency as 25(OH)D ≤ 20 ng/mL (≤50 nmol/L), insufficiency as 25(OH)D 21–29 ng/mL (52.5–72.5 nmol/L) and sufficiency as 25(OH)D 30–100 ng/mL (75–250 nmol/L) [76]. 

Epidemiological studies demonstrated high prevalence of vitamin D deficiency or insufficiency for inhabitants of Europe, China, India, the Middle East and South America [81,82,83,84]. Therefore, supplementation of this vitamin [77] or vitamin D-fortified food intake [85,86] is recommended. 

## 4. Vitamin D Metabolism—Formation of Bioactive Calcitriol

Vitamin D_3_ and D_2_ are biologically inactive forms. They have to be properly metabolized in the skin, liver and kidneys to gain bioactivity and the ability to affect somatic cells. In the skin, 7-dehydrocholesterol is converted into pre-vitamin D_3_ and then into vitamin D_3_, which is subsequently released into the blood. Simultaneously, vitamin D_3_ and D_2_ from the diet are absorbed from the intestinal lumen into the blood. All circulating vitamin D goes to the liver where it is transformed into the 25(OH)D metabolite known as calcidiol. Finally, 25(OH)D goes from the liver to the kidneys and is converted into the biologically active metabolite 1,25(OH)_2_D, known as calcitriol [87]. In order to assess vitamin D status, determination of serum 25(OH)D, instead of 1,25(OH)_2_D, is recommended in clinical practice [88]. The main reason for exclusion of 1,25(OH)_2_D as a diagnostic tool is its short half-life of 6–8 h, a fact that results in daily fluctuations in its serum concentration. Comparatively, the 25(OH)D half-life is 3 weeks. Moreover, in different disease states as well as extraordinary physiological states (e.g., pregnancy), elevated 1,25(OH)_2_D can occur despite actual vitamin D deficiency. Greater costs and difficulty in 1,25(OH)_2_D compared to 25(OH)D determination are further weaknesses [88]. The results of a recent clinical report, however, suggest that serum free and serum bioavailable 25(OH)D, but not total serum 25(OH)D, are the most appropriate and reliable markers for assessing vitamin D status [89].

## 5. Favorable Immunomodulatory Effects of Calcitriol in Autoimmune Diseases

The immunomodulatory effect of calcitriol is based on a genomic response and its ability to modify gene transcription. From the point of view of autoimmune diseases, the most important role of this vitamin D metabolite is its ability to downregulate all mechanisms connected with adaptive immunity and induce immunological tolerance as well as an anti-inflammatory effect [90]. The detailed immunomodulatory effects of 1,25(OH)_2_D on immune cells are presented in Table 1.

Calcitriol enhances the maturation of monocytes into macrophages, but it simultaneously reduces their ability to present antigens to T cells by decreasing the expression of superficial histocompatibility complex MHC-II [91,92]. It also impairs dendritic cell (DC) maturation, which results in the formation of tolerogenic DCs with no surface MHC molecules that are thus unable to present antigens [95,96,97,98,101,102]. Impairment of antigen presentation by antigen-presenting cells (APCs) leads to T cell anergy (lack of response) that inhibits and/or impairs B cell proliferation, differentiation into plasma cells, formation of memory B cells and production of immunoglobulins, including autoantibodies [114,115,116]. Further, calcitriol promotes CD4^+^ T cell differentiation into Th 2 and regulatory T (Treg) cells and reduces Th 1 and Th 17 cell production. This change decreases the Th1/Th2 ratio [107,108,109,110]. Vitamin D also affects cytokine production. It stimulates immune cells to release anti-inflammatory cytokines, such as IL-4, IL-10 and TGF-β, and simultaneously decreases pro-inflammatory cytokine production, including IL-1β, IL-6, IL-12, IL-17, IL-22, TNF-α and IFN-γ [92,95,101,106,109,110,113].

The immunomodulatory effect of calcitriol described above, namely promoting the induction of immune tolerance and T cell anergy, impairing B cells activity and antibody production as well as reducing the inflammatory response, suggests a therapeutic potential for vitamin D in autoimmune diseases (including T1DM). Vitamin D probably plays an important role in reducing the risk of autoimmune diseases and alleviates the disease course.

## 6. Vitamin D Status in T1DM Patients

Preclinical studies of vitamin D action on insulin secretion, insulin action, inflammatory processes and immune regulation, along with evidence of an increase of hypovitaminosis D worldwide, prompted several epidemiological, observational and supplementation clinical studies that investigated potential biological interactions between hypovitaminosis D and diabetes [118]. 

Numerous researchers clearly demonstrate the association between vitamin D and T1DM (Table 2). Swedish studies carried out on 459 T1DM patients aged 15–34 demonstrated that the 25(OH)D concentration at diagnosis was significantly lower compared to the control group. Additionally, diabetic men had a lower 25(OH)D level than diabetic women [119]. Australian children and adolescents with T1DM also had significantly lower serum vitamin D concentration compared to healthy individuals [120]. Similar results were reported by Daga et al. and Federico et al. in Indian and Italian, respectively, case control studies. Specifically, T1DM patients had significantly lower 25(OH)D level and higher prevalence of vitamin D deficiency compared to controls [121,122]. The results of the Bener et al. study conducted on 170 Qatari children under 16 years of age demonstrated that vitamin D deficiency occurs significantly more often in patients with T1DM than in healthy individuals [123]. A similar conclusion was presented by Rasoul et al. as they observed higher frequency of vitamin D deficiency and insufficiency in Kuwaiti children with T1DM than in their healthy peers [124]. The problem of inadequate vitamin D concentration is also common among Swiss children and adolescents with T1DM; 60.5% of 129 examined patients had vitamin D deficiency and 26.4% had vitamin D insufficiency [125]. However, Reinert-Hartwall et al. did not find a significant difference in the 25(OH)D level between β-cell autoantibody positive and negative, non-diabetic children, and vitamin D status was not associated with *FOXP3* gene expression on CD4^+^ T cells responsible for Treg cell production [126].

## 7. Vitamin D Status and the Risk of T1DM

Although the problem of vitamin D deficiency in patients with T1DM is widely known, the cause and effect relationship has yet to be fully explored. It is not fully clear whether an inadequate vitamin D concentration is a T1DM trigger or a consequence of the disease. The available literature suggests both possibilities; vitamin D status is a strong environmental risk factor for T1DM as well as a consequence of physiological and behavioral changes that result from disease. The impact of vitamin D on T1DM risk appears to be dependent on the stage of life. Results regarding the association between prenatal exposure of fetus to vitamin D measured by maternal 25(OH)D level, vitamin D status or vitamin D-fortified food intake in pregnancy and T1DM risk in the offspring are inconclusive. Sørensen et al. demonstrated a significantly lower gestational 25(OH)D level in Norwegian mothers of children who developed T1DM within the first 15 years of life than in those who remained non-diabetic. This study also revealed a significant trend towards a higher risk for T1DM in offspring with lower gestational levels of vitamin D [127]. Moreover, the results of the D-tect study conducted by Jacobsen et al. showed a significant 1.5-to-2-fold higher risk of T1DM in males (but not females) aged up to 14 years old whose mothers did not eat vitamin D-fortified margarine during pregnancy compared to those whose mothers did eat it [128]. Miettinen et al., however, found no significant difference in 25(OH)D level and vitamin D status in pregnancy between Finnish mothers of children who developed T1DM up to the seventh year of life compared to controls [129]. Similarly, the results of a meta-analysis of observational studies conducted by Dong et al. showed no significant association between total vitamin D or cod liver oil intake by pregnant women and the risk of T1DM in their offspring [130]. The 25(OH)D level in newborns at birth was not associated with T1DM risk up to tenth year of life, and the difference in the 25(OH)D level at birth between T1DM cases and controls was insignificant [131]. Further studies are needed to clearly establish whether or not prenatal exposure to vitamin D affects T1DM risk in later life.

Supplementation with vitamin D in infancy, however, appears to have a more pronounced effect on the risk of T1DM than prenatal exposure to this vitamin. The results of meta-analysis conducted by Zipitis et al. that included observational studies mainly from the EURODIAB project suggests that vitamin D supplementation at this stage of life may decrease the risk of T1DM in later life [132]. The same conclusion was made by Dong et al. [130]. In both analyses, T1DM risk in individuals who received vitamin D supplementation in infancy was reduced almost 1.5-fold [130,132]. Moreover, Jacobsen et al. reported that exposure to vitamin D-fortified margarine during the first year of life (probably through breastfeeding) reduced the risk of T1DM in males (but not females) aged up to 14 years old by 2–7.5 times [128]. 

Stene and Joner demonstrated, however, that the timing of vitamin D administration is crucial to achieve the beneficial effect. Infants supplemented with vitamin D between 7 and 12 months of age had a 1.8-fold lower T1DM risk than those supplemented from birth until 6 months of age [133]. This finding is probably due to the fact that adaptive immunity mechanisms become competent during the second 6 months of life. Thus, during the first 6 months of life, vitamin D does would have little to no regulatory effect on their action [134]. Vitamin D supplementation dosage and duration in infancy were not considered in the majority of studies included in meta-analyses mentioned above. Only Hyppönen et al. demonstrated that regular supplementation with the recommended daily vitamin D dose (2000 IU for Finnish infants) during the whole infancy period reduced the risk of T1DM (4-to-5-fold) within the first year of life compared to regular supplementation with lower doses [135]. 

The results of follow-up studies conducted on children at different ages suggest no association between pre-diagnostic vitamin D status and T1DM onset in later life. Although Raab et al. demonstrated a significantly lower 25(OH)D level and higher prevalence of vitamin D deficiency in German children with islet auto-antibodies (IAab-positive) compared to IAab-negative controls, they found no association between vitamin D deficiency and T1DM progression in IAab-positive children over an almost 6-year observation period [136]. There was no significant difference in pre-diagnostic 25(OH)D level between Finnish children who developed T1DM and those who remained healthy as well as no relationship between the 25(OH)D level and risk of T1DM [137]. Similarly, Simpson et al. found no significant association between pre-diagnostic vitamin D status and 25(OH)D level in US American children and risk of IA or progression to T1DM [138].

In young adults, however, vitamin D status appears to exert a clear impact on T1DM risk. A follow-up study carried out by Gorham et al. on US American military service members showed significantly lower 25(OH)D levels in individuals who during an average of 1 year developed insulin-requiring DM compared to age-matched controls. Moreover, they found a significant trend towards a higher risk of insulin-requiring DM in individuals with lower pre-diagnostic serum 25(OH)D [139]. Another study conducted with a similar group (US American active-duty military personnel) by Munger et al. supported Gorham’s et al. findings, but only for non-Hispanic whites [140]. They demonstrated significantly lower T1DM risk for those individuals with a pre-diagnostic 25(OH)D level ≥100 nmol/L compared to those with a level <75 nmol/L. They also observed a significant trend towards a higher T1DM risk in individuals with lower 25(OH)D levels. Finally, there was no significant association between the 25(OH)D level and T1DM risk in non-Hispanic blacks or Hispanics [140]. 

The results presented above clearly demonstrate that vitamin D status can be considered an environmental risk factor of T1DM, especially at some life stages, e.g., infancy. Simultaneously, there is unequivocal evidence that the widespread problem of vitamin D deficiency in T1DM patients may be a consequence of physiological and behavioral changes that result from the disease. Thrailkill et al. reported enhanced vitamin D binding protein excretion in urine from T1DM patients, and this finding suggests that the disease-induced disrupted vitamin D metabolism can increase the risk of vitamin D deficiency in patients [141]. Moreover, reduced outdoor and sunlight exposure, as declared by T1DM patients, may also contribute to vitamin D deficiency in this group of people [120]. It can therefore be assumed that an insufficient vitamin D concentration increases the T1DM risk, which in turn exacerbates the 25(OH)D deficiency. Table 3 presents follow-up studies regarding the association between pre-diagnostic vitamin D status and T1DM risk.

## 8. Vitamin D in T1DM Treatment

Many interventional studies and randomized controlled trials established positive clinical effects in patients with T1DM. Insulin therapy supplemented with different forms of vitamin D—cholecalciferol, alfacalcidiol and calcitriol—improve the preservation of residual pancreatic β-cells function in T1DM patients [142,143,144,145,146]. A significantly higher level of fasting C-peptide (FCP) and/or lower needed daily insulin dose (DID) was observed in supplemented groups. Li et al. noted, however, that the positive effect occurred only in latent autoimmune diabetes of adults (LADA) patients with a diabetes duration ≤12 months [144]. Moreover, Pitocco et al. showed that the decreased insulin need was only temporary (up to 6 months) [146]. Males appear to be more sensitive to alfacalcidiol, since Ataie-Jafari et al. observed a stronger increase in FCP and decrease in DID in males compared to females [145]. Mishra et al. presented a trend, although insignificant, toward a lower decline in residual pancreatic β-cell function in supplemented patients [147]. Additionally, Federico et al. demonstrated significant inhibition of auto-aggression and protective effect on pancreatic β-cells function in patients supplemented with calcidiol. Decreased reactivity of peripheral blood mononuclear cells against glutamic acid decarboxylase and pro-insulin as well as stable FCP level was observed in this group [148]. 

The results of interventional studies conducted by Giri et al., Dehkordi et al. and Bogdanou et al. revealed that cholecalciferol adjuvant therapy improves glycemic control in T1DM patients. A significant decrease in HbA1C (glycated hemoglobin) level occurred after 3 months of treatment with cholecalciferol at different doses [143,149,150]. This form of vitamin D also prevents micro- and macrovascular complications related to chronic elevated blood glucose in diabetic patients [150] and improves endothelial function [151]. 

Interventional studies and randomised controlled trials proved that insulin therapy supplemented with cholecalciferol has a protective immunological effect in both patients with recent-onset T1DM as well as those with a longer duration of diabetes. Gabbay et al. observed a significant increase in Treg cells in patients supplemented with 2000 IU of cholecalciferol daily for 12 months [152]. A similar effect was reported by Bogdanou et al., who administrated 4000 IU of cholecalciferol daily for 3 months to T1DM patients. An increased Treg cell percentage, however, was only observed in males [143]. Cholecalciferol not only enhances the number of Treg cells, but it also improves their suppressor function. Indeed, Treiber et al. demonstrated this vitamin D_3_ capacity in a randomised controlled trial [153]. After 12 months of daily 70 IU/kg cholecalciferol supplementation, the supplemented group had a significantly increased Treg cell suppressive capacity compared to the placebo group [153].

There are also studies that indicate no significant role for vitamin D in the treatment of T1DM patients. Bizzarri et al. and Walter et al. conducted randomised double-blinded placebo-controlled trials in recent-onset patients with T1DM. They demonstrated no protective effect of daily supplementation with 0.25 µg calcitriol for 18–24 months on pancreatic β-cell function [154,155]. Perchard et al., in their 24-month interventional study that involved one-off supplementation of 100,000 or 160,000 IU of cholecalciferol, indicated no effect on glycemic control in children with T1DM [156]. 

Detailed information on the interventional studies cited above are presented in Table 4, and Table 5 contains detailed information on randomised controlled trials. 

It should be noted that the vitamin D response index that results from an individual molecular response to vitamin D supplementation could explain the discrepancies between studies and could be used for stratification of future study cohorts.

## 9. Conclusions

T1DM is a chronic autoimmune disease associated with pancreatic β-cell degeneration that results in the inability to produce insulin and the consequent need for exogenous insulin administration. It is a significant global health problem, since the incidence of this disorder is increasing worldwide, and health complications are very serious, affect many organs and bring high economic expenditures for patients and countries. Thus, this disease is a significant challenge for healthcare systems and raises the need to search for low-cost adjuvant therapies in diabetic patients. 

The potential of vitamin D as a therapy for T1DM patients is apparent. Its immunomodulatory properties, which promote the induction of immune tolerance and T cell anergy, impair B cell activity and antibody production as well as reduce the inflammatory response (Table 1), exert beneficial effects for prevention (Table 2 and Table 3) and treatment of T1DM (Table 4 and Table 5). However, it should be emphasised that many clinical intervention studies that used various forms of vitamin D for the prevention of T1DM or in patients already affected with the disease have been disappointing. 

The alarming fact is that epidemiological studies have demonstrated a worldwide high prevalence of vitamin D deficiency or insufficiency. The problem of vitamin D deficiency is widely known, especially in patients with T1DM. Insufficient vitamin D is not only a consequence of disease, but it seems to be primarily a strong dietary trigger. Adequate vitamin D supplementation in childhood appears to exert a protective effect and decrease the risk of T1DM later in life. Thus, recommendations on daily vitamin D intake and supplementation should be followed by all age groups, particularly infants and children. 

Concern for the optimal status of vitamin D, especially in early life, is an important preventative element of T1DM. Nevertheless, additional studies are needed to establish appropriate vitamin D dosage and form (cholecalciferol, alfacalcidiol or calcitriol) used as an adjuvant therapy with insulin treatment, adjusted for individual needs of diabetic patients (age, degree of vitamin D deficiency or insufficiency, duration of diabetes and current insulin needs). Importantly, vitamin D supplementation up to 10,000 IU per day is a safe and tolerable upper level intake for adults (determined by the Unites States Institute of Medicine). This finding opens the door for treatment trials with vitamin D levels high enough to effectively reduce T1DM incidence and complications. Recent reports indicate new directions for further research on this issue and suggest that future recommended daily vitamin D intake in various human populations, as well as supplementation dosage in patients with T1DM, may depend on polymorphisms in genes involved in the vitamin D metabolic pathway and on the personal vitamin D response index.

## Figures and Tables

**Table 1:** Immunomodulatory effect of 1,25(OH)_2_D.

Immune Cell Type	Calcitriol-Induced Effect	References
Macrophages	↓ Pro-inflammatory IL-1β, IL-6, TNF-α ↑ Anti-inflammatory IL-10 ↓ Antigen presentation → T cells anergy	[91,92,93,94,95]
Dendritic cells	↓ Pro-inflammatory IL-12, TNF-α ↑ Anti-inflammatory IL-10, TGF-β ↓ DCs differentiation, maturation, activation → tolerogenic DCs ↓ Antigen presentation→ T cells anergy ↑ Treg	[96,97,98,99,100,101,102,103]
CD4^+^ T cells	↓ Th 1, Th 17, Th1/Th2 ↓ Pro-inflammatory IFN-γ, IL-17, Il-22 ↑Th 2, Treg ↑ Anti-inflammatory IL-4, IL-10	[104,105,106,107,108,109,110]
CD8^+^ T cells	↓ Hyperactivation ↓ Pro-inflammatory IFN-γ, TNF-α	[111,112]
B cells	↑ Anti-inflammatory IL-10 ↓ B cells proliferation, differentiation into plasma cells ↓ Formation of memory B cells ↓ Immunoglobulins, including auto-antibodies	[113,114,115,116,117]

**Table 2:** Vitamin D and type 1 diabetes mellitus: observational case-control studies.

Place of Study	Cases and Controls	25(OH)D Level at Diagnosis	Vitamin D Deficiency	Significant Findings	References
Sweden	Age: 15–34 y Cases: 459 Controls: 208	Cases: 82.5 ± 1.3 nmol/L ♂: 77.9 ± 1.4; ♀: 90.1 ± 2.4 nmol/L Controls: 96.7 ± 2.0 nmol/L		Significantly lower 25(OH)D level in cases than in controls. Significantly lower 25(OH)D in diabetic men than women.	[119]
Australia	Age: pediatric Cases: 56 Controls: 46	Cases: 78.7 (71.8–85.6) nmol/L Controls: 91.4 (83.5–98.7) nmol/L		Significantly lower 25(OH)D level in cases than in controls.	[120]
India	Age: <25 y Cases: 72 Controls: 41	Cases: 7.88 ± 1.2 ng/mL Controls: 16.64 ± 7.83 ng/mL	Cases: 91.1% Controls: 58.5%	Significantly lower 25(OH)D level and higher prevalence of vitamin D deficiency in cases than in controls.	[121]
Italy	Age: pediatric Cases: 82 Controls: 117	Cases: 54.4 ± 27.3 nmol/L Controls: 74.1 ± 28.5 nmol/L	Cases: 48.8% Controls: 17.9%	Significantly lower 25(OH)D level and higher prevalence of vitamin D deficiency in cases than in controls.	[122]
Qatar	Age: <16 y Cases: 170 Controls: 170		Cases: 90.6% Controls: 85.3%	Significantly higher prevalence of vitamin D deficiency in cases than in controls.	[123]
Kuwait	Age: pediatric Cases: 216 Controls: 204		Cases: total 99% Deficiency: 84% Insufficiency: 15% Controls: total 92% Deficiency: 77% Insufficiency: 15%	Significantly higher prevalence of vitamin D deficiency and vitamin D (deficiency + insufficiency) in cases than in controls.	[124]
Finland	Age: pediatric Cases: 35 Controls: 80	Cases (β-cell Aab+): 70.6 ± 20.8 nmol/L Controls (β-cell Aab−): 65.7 ± 19.4 nmol/L		No significant difference in 25(OH)D level in cases and controls.	[126]

♂—males; ♀—females; Aab+: autoantibodies-positive; Aab−: autoantibodies-negative.

**Table 3:** Vitamin D status and the risk of type 1 diabetes mellitus: prospective cohort follow-up studies.

Place of Study	Study Group	Clinical Findings	Conclusions	References
Norway	Age: ≤15 y Cases: 109 Controls: 219 Follow-up: 15 y	Maternal 25(OH)D level (37 week of pregnancy): Cases: 65.8 ± 26.5 nmol/L; Controls: 73.1 ± 27.2 nmol/L Adjusted OR of T1DM for 25(OH)D level [nmol/L]: ≤54 vs >89: 2.38 (95% Cl 1.12–5.07) >54 and ≤69 vs >89: 1.78 (95% Cl 0.85–3.74) >69 and ≤89 vs >89: 1.35 (95% Cl 0.63–2.89) P for trend (continuous): 0.031	Significantly lower gestational 25(OH)D level in mothers of children with T1DM than in those of healthy children. Significant trend toward a higher risk of T1DM in the offspring with lower levels of vitamin D during pregnancy.	[127]
Finland	Age: ≤7 y Cases: 343 Controls: 343 Follow-up: 17 y	Maternal 25(OH)D level (1st trimester of pregnancy): Cases: 43.9 ± 16.9 nmol/L; Controls: 43.5 ± 16.6 nmol/L Maternal vitamin D status (*p* = 0.88): deficiency: cases (*n* = 33), controls (*n* = 32) insufficiency: cases (*n* = 208), controls (*n* = 208) sufficiency: cases (*n* = 85), controls (*n* = 90) optimal: cases (*n* = 17), controls (*n* = 13)	No significant difference in maternal 25(OH)D level and vitamin D status in pregnancy between cases and controls.	[129]
Italy	Age: ≤10 y Cases: 67 Controls: 236 Follow-up: 10 y	Geometric mean of 25(OH)D level at birth: Cases: 1.42 (0.80–3.00); Controls: 1.81 (0.96–3.40) Adjusted OR of T1DM up to 10 y of age (0.78; 95% Cl 0.56–1.10) for each unit increment in log vitamin D	No association between 25 (OH)D level at birth and risk of T1DM up to 10 years of age.	[131]
Finland	Age: ≤1 y Cases: 81 Controls: 10,285 Follow-up: first year of life	Adjusted RR of T1DM for vitamin D supplementation: regular vs no supplement. (0.12; 95% Cl 0.03–0.51) irregular vs no supplement. (0.16; 95% Cl 0.04–0.74) regular recommended dose vs regular less than recommended doses (0.22; 95% Cl 0.05–0.89)	Significantly reduced risk of T1DM in infants supplemented with vitamin D within the first year of life. Higher protective effect provided by the recommended dose of vitamin D (2000 IU).	[135]
Germany	Age: pediatric Cases: 352 IA, including 244 T1DM Controls: 406 Follow-up: 5.8 y	25(OH)D level within 2 y of IAab seroconversion: Cases: 59.9 ± 3.0 nmol/L; Controls: 71.9 ± 1.5 nmol/L Vitamin D deficiency: Cases: 39.8%; Controls: 28.3% Cumulative incidence of T1DM at 10 y after IAab seroconversion: children with vitamin D deficiency: 51.8% children with vitamin D sufficiency: 55.4%	Significantly lower 25(OH)D level and higher prevalence of vitamin D deficiency in IAab-positive children than IAab-negative controls. No association between vitamin D deficiency and progression to T1DM in IAab-positive children.	[136]
Finland	Age: pediatric Cases: 126 Controls: 126 Follow-up: 4.5–6 y	Median 25(OH)D level of multiple collected samples before diagnosis: Cases: 66.6 nmol/L (14.0–262.8) Controls: 67.4 nmol/L (19.9–213.0) Correlation between 25(OH)D level and: age at seroconversion to IAab-positivity (*p* = 0.79) T1DM onset (*p* = 0.13)	No significant difference in 25(OH)D level between cases and controls. No association between 25(OH)D level and risk of T1DM.	[137]
USA	Age: pediatric Cases: 198 IA, including 90 T1DM Controls: 2644 Follow-up: 18 y	Adjusted HR of T1DM for intake of vitamin D and: risk of IA (1.13; 95% Cl 0.95, 1.35; *p* = 0.18) progression to T1DM (1.12; 95% Cl 0.91, 1.86; *p* = 0.15) Adjusted HR of T1DM for 25(OH)D level and: risk of IA (1.12; 0.95% Cl 0.88, 1.43; *p* = 0.36) progression to T1DM (0.91; 95% Cl 0.68, 1.22; *p* = 0.54)	No association between vitamin D intake and 25(OH)D levels throughout childhood and risk of IA or progression to T1DM.	[138]
USA	Age: 17–35 + Cases: 1000 Controls: 1000 Follow-up: 10 y	25(OH)D level 1 y (1 mo–10 y) before diagnosis: Cases: 62.2 ± 31.8 nmol/L; Controls: 72.5 ± 33.0 nmol/L Adjusted OR of insulin-requiring DM for 25(OH)D level [nmol/L]: <43 vs ≥100: 3.5 (95% Cl 2.0–6.0) 43–59 vs ≥100: 2.5 (95% Cl 1.5–4.2) 60–77 vs ≥100: 0.8 (95% Cl 0.4–1.4) 78–99 vs ≥100: 1.1 (95% Cl 0.6–2.8) P for trend (continuous): <0.001	Significantly lower 25(OH)D level in cases than in controls. Significant trend toward a higher risk of insulin-requiring DM in individuals with lower pre-diagnostic serum 25(OH)D.	[139]
USA	Age: ≥ 20,6 ± 4,0 Cases: 310 Controls: 613 Follow-up: 5.4 y	Adjusted RR of T1DM for 25(OH)D level (nmol/L): (non-Hispanic whites) <75: 1.0 (referent) 75–99.9: 0.60 (95% Cl 0.38–0.97) ≥100: 0.56 (95% Cl 0.35–0.90) P for trend (continuous): 0.03 Adjusted RR of T1DM for 25(OH)D level (nmol/L): 31–76.9 (referent): 1 77–88.6 vs 31–76.9: 0.47 (95% Cl 0.25–0.88) 88.7–100.6 vs 31–76.9: 0.45 (95% Cl 0.24–0.82) 100.7–114.0 vs 31–76.9 7: 0.3 (95% Cl 0.16–0.58) 114.1–210.7 vs 31–76.9: 0.47 (95% Cl 0.26–0.86) P for trend (continuous): 0.008	Significantly lower risk of T1DM in non-Hispanic whites with pre-diagnostic 25(OH)D level ≥ 100 nmol/L than < 75 nmol/L. No significant association between 25(OH)D level and the risk of T1DM in non-Hispanic blacks and Hispanics. Significant trend toward a higher risk of T1DM in individuals with lower 25(OH)D level.	[140]

IAab—islet autoantibodies; IA—islet autoimmunit.

**Table 4:** Therapeutic role of vitamin D in patients with type 1 diabetes mellitus: interventional studies.

Treatment Duration	Study Group	Supplementation Dosage *	Significant Changes in Biochemical Parameters	Clinical Findings	References
12–24 months	N = 31; Age:15.7 ± 1.4 y 25(OH)D <37.5 nmol/L Dd: 6.8 ± 3.5 y	Cholecalciferol 1000–2000 IU/d	↑ 25(OH)D, InRHI ↓ MCP-3, EGF, TNF-β, IL-10	Significant improvement in endothelial function. Significant decrease in urinary inflammatory cytokines.	[151]
12 weeks	N = 22	Cholecalciferol 4000 and 10,000 IU/d	↑ Glycemia standard deviation ↓ DID	Significant improvement in glycemic variability, lower insulin needs and lower frequency of hypoglycemia.	[142]
3 months	N = 73 Age: 7.7 ± 4.4 y 25(OH)D < 75 nmol/L Dd: no data	Cholecalciferol 6000 IU/d (in vit. D-deficient patients) or 400 UI/d (in vit. D-insufficient patients)	↑ 25(OH)D ↓ HbA1C	Significant improvement in glycemic control.	[149]
12 weeks	N = 30; Age: 5–15 y 25(OH)D < 75 nmol/L Dd: 3.1 ± 1.3 y	Cholecalciferol 50,000 UI/week	↑ 25(OH)D, IGF-1 ↓ HbA1C	Significant improvement in glycemic control and prevention of its related micro- and macrovascular complication.	[150]
6 months	N = 39 Age: 44 (33–52) y Dd: 12.3 (2.8–24.5) y	Cholecalciferol 4000 IU/d for 3 mo followed by placebo for 3 mo or in the sequential alternative	↑ 25(OH)D, FCP ↓ HbA1C, DID ↑ Tregs (♂)	Significant improvement in glycemic control and preservation of pancreatic β-cells function In males, increase in regulatory T cells.	[143]
6 months	N = 15 + 15 Age: 6–12 y Dd: 1–2 y	I: Cholecalciferol 2000 IU/d + Ca 25 mg/kg/d II: Unsupplemented	↓ Mean and monthly decrease in SCP in supplemented group, insignificant	Trend toward lesser decline of residual pancreatic β-cells function in supplemented patients.	[147]
12 months	N = 8 + 7 Age: 12 ± 0.9 25(OH)D < 20 ng/mL Dd: 0.7 ± 0.2 y	I: Calcidiol 10 µg/d to reach 50–80 ng/mL (reached after 2 mo) II: Unsupplemented	↓ reactivity of PBMCs against GAD and pro-insulin in supplemented group FCP stable at 12 mo	Significant inhibition of autoagression and protective effect on pancreatic β-cells function in supplemented patients.	[148]
24 months	N = 42 Age: 12.7 ± 3.1 y 25(OH)D < 50 nmol/L Dd: 4.8 ± 3.3 y	Cholecalciferol 1 dose of 1000,000 IU (2–10 y) or 1 dose of 160,000 IU (>10 y)	No difference in mean HbA1C level at 3 or at 12 months before and after treatment.	No effect on glycemic control.	[156]

* Plus insulin therapy. Dd—diabetes duration; InRHI—reactive hyperemia index; MCP-3—monocyte chemotactic protein-3; EGF—epidermal growth factor; TNF-β—tumor necrosis factor beta; IL-10—interleukin 10; DID—daily insulin dose; HbA1C—glycated hemoglobin; IGF-1—insulin like growth factor 1; FCP—fasting C-peptide; Tregs—regulatory T-cell; SCP—stimulated C-peptide; PMBCs—peripheral blood mononuclear cells; GAD—glutamic acid decarboxylase.

**Table 5:** Therapeutic role of vitamin D in patients with type 1 diabetes mellitus: randomized controlled trials (RCT).

Study Design	Study Groups	Supplementation Dosage *	Significant Changes in Biochemical Parameters	Clinical Findings	References
RCT, DB, PC 12 months	N = 30 (14 + 15) Age:12(11–17.5) y Dd: 61 ± 20 d	I: Cholecalciferol 70 IU/kg/d II: Placebo	↑ Suppressive capacity of Tregs in suplemmented group in contrast to placebo group	Significant improvement in suppressor function of Tregs in supplemented patients	[153]
Prospective RCT 12 months	N = 35 (18 + 17) Age: 38.5 ± 12.5 y Dd: 1.0 (0.1–4.0) y LADA patients	I: Unsupplemented II: Alfacalcidiol 0.5 µg/d	I: ↓ FCP, 22% had stable FCP II: 70% had stable or ↑ FCP	Significant improvement in preservation of pancreatic β-cells function in supplemented patients, but only with diabetes duration ≤ 12 mo	[144]
RCT 12 months	N = 67 (34 + 33) Age: 13.6 ± 7.6 y Dd: <4 weeks	I: Calcitriol 0.25 µg/d II: Nicotinamide 25 mg/kg/d	I: ↓ DID, but only at 3 and 6 mo In both groups no changes in FCP and HbA1C at 12 mo	Modest effect on residual pancreatic β-cells function and only temporarily reduction of insulin dose in calcitriol- supplemented patients.	[146]
RCT, DB, PC 18 months	N = 38 (19 + 19) Age: 13.5 ± 5.1 y Dd: 2.2 ± 1.2 mo	I: Cholecalciferol 2000 IU/d II: Placebo	↑ Chemokine ligand 2 at 12 mo ↑ SCP and Tregs % at 12 mo ↓ Progression to undetectable FCP at 18 mo	Protective immunological effect and slowing down the decline of residual pancreatic β-cells function in supplemented patients.	[152]
RCT, SB, PC 6 months	N = 54 (29 + 25) Age: 10.2 ± 2.5 y Dd: 44 ± 14 d	I: Alfacalcidiol 0.5 µg/d II: Placebo	↑ FCP ↓ DID Stronger response in males.	Improvement in preservation of pancreatic β-cells function in supplemented patients with a stronger effect in males.	[145]
RCT, DB, PC 24 months	N = 27 (15 + 12) Age: 18 (11–35) y Dd: <12 weeks	I: Calcitriol 0.25 µg/d II: Placebo	No differences in FCP, HbA1C and DID between groups.	No protective effect on pancreatic β-cells function in supplemented patients.	[154]
RCT, DB, PC 18 months	N = 40 (22 + 18) Age: 31.4 ± 6.8 y Dd: 35 d	I: Calcitriol 0.25 µg/d II: Placebo	No differences in FCP and DID between groups.	No protective effect on pancreatic β-cells function in supplemented patients.	[155]

* Plus insulin therapy; Dd—diabetes duration; DB—double-blinded; SB—single-blinded; PC—placebo-controlled; HbA1C—glycated hemoglobin; FCP—fasting C-peptide; SCP—stimulated C-peptid.
